# Highs and lows: patterns of use, positive and negative effects of benzylpiperazine-containing party pills (BZP-party pills) amongst young people in New Zealand

**DOI:** 10.1186/1477-7517-4-18

**Published:** 2007-11-19

**Authors:** Rachael A Butler, Janie L Sheridan

**Affiliations:** 1 School of Pharmacy, University of Auckland, Private Bag 90219, Auckland, New Zealand

## Abstract

**Background:**

This study aimed to investigate patterns and context of use of BZP-party pills, function of use, and positive and negative effects experienced by a sample of New Zealand young people who had used the products.

**Methods:**

A qualitative study comprised of semi-structured interviews and group discussions.

**Results:**

The sample included 58 young people aged 17–23 years who had used BZP-party pills in the previous 12 months. Young people were using these substances in a range of settings – primarily during weekend social occasions – particularly as part of the dance party culture. They were mostly used for their stimulant properties and to enhance socialisation, and were often taken in combination with other legal and illicit drugs. Young people had suffered a range of physical and emotional negative effects, although none of these was reported as being life-threatening or long-term. Many participants had reduced the frequency with which they used BZP-party pills due to adverse effects. Potentially risky behaviours identified included taking large doses, mixing BZP-party pills with alcohol and other substances, and driving whilst under the influence of BZP-party pills.

**Conclusion:**

Findings suggest that young people in this study were not suffering excessive or dangerous adverse effects. However, potentially risky use of these products raises the issue of the need for developing harm reduction interventions.

## Background

A recent phenomenon to have emerged in New Zealand, and one which has proven to be popular amongst young people, is the use of pills containing benzylpiperazine (BZP), and sometimes also trifluoromethylphenylpiperazine (TFMPP). They may also contain additional substances such as guarana and vitamins.

BZP has been shown to have amphetamine-like effects [1] and, whilst there is no published literature on the effects of TFMPP on humans, research in rats indicates that is has 'MDMA-like' properties [2]. At the time of the study (2006), BZP-containing party pills were legally available for sale in a wide range of retail outlets in New Zealand and could also be purchased via the internet and mobile delivery services. The products, usually known as 'legal party pills' in New Zealand, are mostly sold in either capsule or pill format, and, at the time of the research, retailed for between $10 and $60 per pack.

A 2006 household survey found that one in five people had ever tried BZP-containing party pills, and one in seven had used them in the preceding twelve months. Levels of use were highest among the 18–24 year age range, with around one third of 18–19 year olds and 38% of 20–24 year olds having used legal party pills in the preceding year [3]. Their current popularity in New Zealand, particularly amongst young people, may be related to the relatively restricted and expensive illicit market for drugs such as ecstasy.

In New Zealand, BZP is currently scheduled as a 'Restricted Substance' under the Misuse of Drugs Amendment Act 2005, meaning that it is illegal to sell or supply BZP-party pills to anyone under 18 years, and 'free giveaways' and print/radio/television advertising of the products are banned. However, at the time of writing (November 2007) the New Zealand Government was reviewing the legal status of benzylpiperazine, having announced that it was recommending that the substance be reclassified as a Class C1 drug, thus making it illegal. An announcement regarding any changes in the law is expected in December 2007. The legal status of BZP is also currently being reviewed in the UK, whilst several other countries have already banned it, including Australia and the US.

There is very limited published data on these substances – both within New Zealand and internationally – particularly with regard to use of the drugs by young people [4]. The primary aims of this research were to investigate patterns and context of use of BZP-party pills, function of use, positive and negative effects, and knowledge of safe use amongst young people aged 16–24 years. Whilst not reported here, the study also explored aspects of marketing and supply, and views of use by young people amongst 'key experts' employed in associated sectors (e.g. health, education and the BZP-party pill industry).

The aim of this paper is to describe the patterns and context of BZP-party pill use, and positive and negative effects experienced by the young users of BZP-party pills who took part in this study. A brief synopsis of the data presented here has previously been reported elsewhere [4] – this paper provides a detailed analysis and interpretation of those results.

## Methods

Qualitative research methods were adopted for this exploratory study. Data were analysed utilising a general inductive approach [5], a systematic procedure for analysing qualitative data which draws on grounded theory [6]. In keeping with the study's youth development approach, the research was also aligned with the six principles^i ^of the Youth Development Strategy Aotearoa (YDSA) [7]. Thus, the researchers worked with young people, where possible, throughout the set-up, recruitment and data collection phases^ii^. This included training and working with youth 'fieldworkers' who were employed to recruit participants, developing relationships with youth organisations, and consulting young people on the methods used to attract participants and to collect data. Ethics approval for the study was granted by the University of Auckland Human Participants Ethics Committee (ref 2005/279).

### Recruitment

The study was publicised via flyers and cards (distributed in party pill retail outlets, bars and clubs, tertiary education campuses, and a range of youth and health organisations) and advertisements placed in suitable online and paper-based publications (e.g. student magazine, entertainment websites). 'Snowballing' was also utilised as a recruitment method, with participants referring their friends to the research team. This recruitment approach has shown to be effective in reaching 'hidden' populations, such as drug users [8].

Inclusion criteria for the study were young people aged 16–24 years old who had used BZP-party pills at least once in the last 12 months, and who were willing to give written, informed consent in order to take part. They were excluded if they, or a close friend or family member, were involved in the manufacture, distribution or retail of BZP-party pills. Potential participants were able to indicate their interest in taking part in the research either via text or by calling a Freephone number, and were screened over the telephone to determine their eligibility to participate.

### Data collection

Research participants were given the option of taking part either in an individual interview, a 'friendship interview/group' (made up of two or more young people known to each other), or a general focus group (with participants unknown to one another). Friendship interviews/groups are widely used in research involving young people and can provide opportunities to observe natural social networks, whilst also aiding recruitment and access to youth respondents [9]. This proved to be the case with this study, with the vast majority electing to take part with friends. Given the younger age of participants, a co-facilitator with a youth treatment background was present at interviews/groups which contained 16–18 year olds.

Interviews and group discussions were semi-structured. The key questions were shaped by the need to explore users' experiences (both positive and negative) of a new substance, about which little was known – and were developed with input from the project advisory group and piloted prior to the research commencing. Topics covered included patterns of use (e.g. accessing products, place of use, frequency, dosage etc.), positive and negative effects, general perceptions of the products, knowledge of safe use and legislation, and (where appropriate) use of other substances. Prior to the main discussion, a pre-group/interview questionnaire was given to participants to complete, which collected additional data on BZP-party pill taking behaviour and other substance use over the previous six months. All respondents provided signed, informed consent and received a movie voucher as a 'thank you' for their contribution to the research.

Interviews and groups were conducted between June and September 2006, with each lasting between 1 and 1 1/2 hours. They were held in a range of community locations (e.g. a neighbourhood community centre) and food and (non-alcoholic) refreshments were provided for participants.

### Analysis

Data from the pre-interview/group questionnaires were entered into an excel spreadsheet and descriptive statistics obtained. All interviews and group discussions, except one^iii^, were recorded and transcribed and thematic analysis of the data was undertaken, utilising a general inductive approach [5]. A selection of transcripts was first read through by the lead researcher and a basic coding frame developed. Data coding was undertaken with the aid of computer-assisted qualitative data analysis software (N-VIVO), with two members of the research team coding half the transcripts each. The coding frame was developed and amended with input from both researchers, where appropriate, during this process. To ensure reliability, the coded data were then read through by both researchers and checked for consistency. Analysis of the data was undertaken with additional input from two further researchers with different backgrounds, thus adding another dimension to the analysis process.

## Results

### The sample

Ten individual interviews, 11 paired interviews and 7 groups (range 3–5 respondents) were conducted, and comprised a total of 58 young people (28 female and 30 male). The average age of participants, based on pre-group/interview questionnaire data, was 19.8 years (range 17–23; data missing on 5 cases). The sample was heavily weighted towards New Zealand European respondents (n = 49) and included a mix of secondary school students, university students, full-time or part-time employees, and an at-home mother. The majority of interviews and groups were conducted in two large urban centres in New Zealand.

All but one person had drunk alcohol during the previous six months (data missing on one person). Twenty two reported not having smoked cigarettes and 12 reported having used no illegal drugs during this time. Of those who reported having used illicit drugs, 34 had used cannabis, 30 had used ecstasy, 16 had used LSD (lysergic acid diethylamide), four had used nitrous oxide, and three or fewer young people had used 'speed', GHB (gammahydroxybutyrate), methamphetamine, 'magic mushrooms', and ketamine. (Note: respondents could provide more than one response).

### First time use of party pills

Data from the pre-group/interview questionnaire identified that the mean age of first use of BZP-party pills was 17.4 years (youngest 14 and oldest 22; data missing on one case). Some had used illicit substances prior to BZP-party pills, whereas for others (alcohol aside) their first experience of drug-taking had involved party pills. Most were introduced to BZP-party pills by a friend or relative who had used them previously.

First use of the substances appears to be a seminal event amongst these young people – not least because it was often a negative experience. In some cases, this was because it involved taking a large number of pills in combination with alcohol – often on the recommendation of a friend or peer:

*First time I took party pills I was 20. She *[friend]* took me to *[name of specialist retailer] *and gave me a couple of packets of *[name of product].* I had no idea what I'd taken but I trusted her. Afterwards she told me we'd taken three times the recommended dosage*. [Interview [8]]

This finding again highlights the role of peers and social networks with regard to initiation into adolescent drug use, and the fact that friends are a trusted source of advice and substances [10-12]. However, as evident in the interview extract above, such advice may be ill-informed or unsafe.

Some young people recalled first time use of BZP-party pills positively, as being an encounter with something exciting and new:

*A couple of mates gave me some *[name of product].*They are really weak, but because it was my first time it was fantastic. I danced my arse off all night*. [Interview [19]]

The above two interview extracts also demonstrate the context of use of the pills – i.e. accompanied by extended periods of dancing – and the common finding that users often take many more pills than is 'recommended'. Both have associated risks, including increased likelihood of negative effects in the first example, and potential overheating and dehydration in the second.

### Frequency of use

Data from the pre-group/interview questionnaire showed that nearly half the sample (n = 25) had used them more than once a month in the last six months, with 31 participants having used them once a month or less during this time period.

A number of respondents had reduced the frequency with which they used BZP-party pills. For most, this was due to the negative side-effects of the products:

I: So you don't take them as much as you used to. Do you want to tell me a little bit about that?

R1:*It's okay at the time but the next day just kills you. And even at the time it's good but it's never that great like you always kind of feel a bit....*

R2:*When you first start it you're like "Wow, this is awesome" but then...*

R1:*Yeah, like 3 months, it's not good. It's all, I don't know, it's relentless. You can't stop it or slow down or anything. Say you're trying to sleep but nah.*

R2:*Yeah, I haven't thought about it for a while. You know what they say. Bad kind of outweighs the good*. [Interview [24]]

Others spoke about life changes that had impacted on their frequency of use (e.g. pregnancy, change of job) or highlighted that levels of use fluctuated throughout the year (e.g. increased occasions of use during the summer).

### Amount used

The data in this section need to be interpreted with caution, as the amount of BZP and TFMPP per pill varies considerably between brands (it has been reported that over 120 brands have been available for purchase in New Zealand [13]); it is therefore not possible to calculate the actual amount of these chemicals per individual pill or capsule.

Young people's behaviour varied with regard to the number of party pills they were taking on each occasion. Some were only taking one pill, whereas others were taking up to six. Many were taking more than the 'recommended amount'^iv ^on the packaging, with a number of interviewees reporting that they took around two to three pills per occasion on average. The pre-group/interview questionnaire identified that on the last occasion of use, the average number of pills taken was 2.1, with the majority of young people having taken one to two pills (range one to six).

Where people were taking more than one pill they often split this amount, and spread it over the course of the BZP-party pill-taking occasion. Data from the pre-group/interview questionnaire showed that the median time before the first and subsequent pill(s) taken on the most recent occasion they had used BZP-party pills, was 75 minutes. However, some people had taken all the pills at once. Again, given the variation in amount of BZP and TFMPP across individual pills and brands, this may mean that young people experience a greater degree of intoxication than anticipated. There was also evidence that some young people titrated their amount against the effects received:

*Oh yeah, you don't even think how many you've taken – I just, I'll have like three at first and then if that doesn't last me the night I just take another until I've got the right energy*. [Interview [10]]

Young people generally had a specific pattern of use with regard to amount used – i.e. some people would always (or mostly) only take one pill, whereas others would take three or four on most occasions. This was mostly dependent upon the degree of intoxication they felt comfortable with. However, the amount used was sometimes varied, depending on the strength of the products being consumed and the context of use (e.g. smaller doses if being taken at work):

*I use them as a tool. Firstly it was to get wasted but now I'll take them if I don't want to get too wasted, just take two or three party pills – 100 mgs at a time – I would be awake but it wouldn't get me wasted*. [Interview [4]]

Some young people also reported that they had reduced the number of pills they were taking on each occasion in an attempt to reduce the negative effects of the products.

### Drug form and route of administration

The main route of administration for interviewees was swallowing BZP-party pills in their original form. All participants stated that this was their primary means of ingesting the drug, and few had experimented with other routes of administration.

A small number had snorted BZP- party pills as a means of trying something new, and in the expectation that it would provide a quicker 'hit'. All, however, stated that it was a 'one-off' occurrence due to suffering negative effects (e.g. nose bleeds). Other routes of administration included ingesting BZP powder (commonly known as "hummer") which was wrapped in a cigarette paper and swallowed. A minority had also emptied capsules from BZP-party pill products into drinks and consumed the substance in liquid form. No young people interviewed reported injecting BZP or knowing anybody who had. Whilst IV use has been reported elsewhere [3], it has not been identified as widespread behaviour, nor specifically an issue for young people.

### Places and context of use

BZP-party pill use was mostly a social activity undertaken with friends, with the majority of young people using the substances at night, during the weekend. It was predominantly associated with clubbing, 'raves' and dance parties – although some young people were also taking them in bars or at parties or gatherings in their homes:

*Probably just going out to town I suppose, drinking at home, having a few drinks at home then going to town and then taking the party pills when you get there. Or maybe having a party at home or something, drinking alcohol and then maybe having one party pill or something, just to maybe lift it a bit. That's typical for me I think*. [Interview [7]]

Daytime use of legal party pills was less common. Where this occurred, it was associated with outdoor use (e.g. at the beach) or more 'functional' and/or solo activities such as housework or studying:

*I'll take it when I've got heaps of cleaning to do and I'm not feeling in the mood, if I'm tired. Then it gets done really well and then I'll just go to bed at the normal time at night. I take them at 11 am or midday. I get right into things*. [Interview [22]]

A small number of young people claimed to use party pills in the work place – this was either motivated by a desire to enhance their working day, or to assist in the recovery from previous social activities:

*Also if I'm really really tired and I know that I have to work, I'll take a few before I go to work and that will just keep me awake. And, like, you know, no one knows that you're on them because you're still – it's not like taking P *[methamphetamine] *or something where everyone knows that you're on something*. [Interview [21]]

Some young people had driven after taking party pills, either when travelling to, or returning home, from an all-night event. In some cases, young people reported that they took party pills in place of alcohol when they were the 'designated driver' for the evening. A minority had also driven whilst 'high' on BZP-party pills. Young people generally perceived that driving after taking BZP-party pills was safer than 'drink-driving', both in terms of the risk of an accident and a driving-related conviction:

*Go to town with his mates and get someone to drive, the sober driver might go on to herbals *[party pills] *only, because at least they're in a good mood but they're not drinking*. [Interview [10]]

There is no evidence to support whether BZP-party pills pose a greater or lesser risk to driving ability, for example when compared with alcohol. However, a link has been identified between risk perceptions and impaired driving behaviour – i.e. people who believe there is a strong likelihood of being apprehended, or involved in an accident, are less likely to drive while intoxicated [14].

### Concurrent use of other substances with BZP-party pills

As part of the pre-group/interview questionnaire, respondents were asked 'what other things do you normally take with them?' The substance most commonly cited was alcohol (n = 46 participants). Concurrent use of cannabis (24), ecstasy (20), LSD (5), methamphetamine (4), ketamine (3), GHB (3), nitrous oxide (2)^v ^and magic mushrooms (2) was also reported. Only three people indicated that they did not use anything else with BZP-party pills. (Note: participants could provide more than one response).

The interviews/group discussions revealed that the other substances used in combination with BZP-party pills often had a very specific role to play. Cannabis was most often used during the 'comedown' period to manage the negative effects experienced during the latter stages of the drug-taking episode, and to stimulate sleep and appetite:

*Awful thing about party pills is that you can't sleep like, when I have finally been able to go to sleep it's not been a very nice sleep and doesn't last for long, and so I smoke a shedload of weed when I'm on party pills because I don't know, to try to go to bed*. [Interview [2]]

Some of the young people in the sample who used ecstasy took the drugs in combination – either taking BZP-party pills prior to MDMA as a way of 'kick-starting' the night, or in reverse to prolong the effects of ecstasy:

*Ecstasy wears off more quickly as well, like it's not as long lasting. If you're going out and you want to go out all night, you don't want to just go home at three or four – you want to be up until six, take some party pills to keep going*. [Interview [10]]

As highlighted in the findings from the pre-group/interview questionnaire, alcohol was the most popular substance consumed in combination with BZP-party pills, despite there being a high level of awareness amongst the sample that this was not recommended behaviour (note: this information is often contained on BZP-party pill packaging). Drinking often took place prior to taking the substances, as a part of general socialising or in the 'warm-up' to a big event. After ingesting BZP-party pills, a proportion of the sample reported that they stopped drinking – either because they found alcohol unpleasant or they felt sufficiently intoxicated. Others maintained that the substances increased their capacity to consume large amounts of alcohol without experiencing usual feelings of intoxication, and took BZP-party pills as a 'sobering' device:

*I might have been thinking of taking only two that night but I was feeling quite drunk so I took a third one. And then 45 minutes later I felt a bit sober and stuff and that's fine. And I'm always dancing when I take them as well, which is a really good combination to sober up as well*. [Interview [15]]

Indeed, a number stated that they specifically took party pills when 'drunk' to enable them to carry on socialising. Use of the substances after consuming large amounts of alcohol has a number of risks given the potential for the products to mask actual levels of intoxication and, as evident in the participant's comments above, may result in an increased number of pills consumed.

### Positive effects of use

A number of themes were identified when respondents were asked to describe what they viewed as the positive effects of BZP-party pill use. A key positive association with the products was their stimulant properties, with young people highlighting that use of the substances enabled them to stay awake for longer to undertake a wide range of activities. These included dancing, general socialising, work, outdoor pursuits and study:

*Oh it was amazing. I was just studying and remembering everything that I studied, just sort of going and going like a machine. I've never studied that well so I was impressed with myself at the time*. [Interview [3]]

*By the time I get to the weekend I am pretty tired and I've got to make the most of the time that I have with my friends a lot of the time. I make the most of going out and dancing so I don't get tired because I enjoy that part of my life so much. Energy *[party] *pills give me the opportunity to make the most of it for as long as I can*. [Interview [19]]

Many users reported that BZP-party pills made them feel confident and relaxed in social situations, and thus the products facilitated social interactions. As a result, existing friendships were strengthened and new alliances were sometimes formed:

*With party pills, um, I want to talk a lot, you've probably heard that before. And we don't want to dance, we just sit down and talk for hours and really enjoy, really get into it and share our deepest and darkest secrets and it's great*. [Interview [12]]

Several young people spoke about the nature of the 'high' when considering positive effects of BZP-party pill use. They described the *"buzz" *experienced during the 'peak' of the drug-taking occasion, alongside a range of sensory and mind altering effects. A number of young women also identified weight loss as a positive outcome of use. Some were using party pills specifically as a weight loss device, whilst others reported this as an added 'bonus' when socialising:

*Me and *[name of friend]*have got the same figure and stuff. We have little podgy bits around here so we get the permanent creases. But after party pills you're all sunken in and you don't have those. It makes you so much more confident*. [Interview [25]]

Such accounts of positive effects are in keeping with the experiences of users of other substances, particularly ecstasy [15,16].

### Adverse/negative effects

A wide range of adverse or negative effects of BZP-party pills were self-identified by respondents, although it should be noted that it is reasonably difficult to attribute many of these to BZP-party pills alone. A combination of BZP-party pill consumption, concurrent use of alcohol and other substances, extended periods of exertion, lack of sleep, and dehydration may all contribute to these negative effects.

The adverse effects identified by the study participants can be divided into two categories: *immediate effects *(i.e. those that were experienced whilst intoxicated) and *after-effects *(experienced once the effects of the drugs had worn off, commonly referred to as the 'comedown' period).

#### Immediate effects (effects while intoxicated)

The negative immediate effects of the substances, as described by respondents, were predominantly physical. Those most commonly cited included: headaches, vomiting and nausea, dehydration/inability to quench thirst, racing heart, sore or shaking body, loss of appetite, trembling mouth or jaw, and an inability to urinate. Others mentioned less often included impaired sexual performance, dizziness, sore eyes, stomach pains, teeth grinding, increased rates of smoking, and bleeding nose (from snorting). Most young people interviewed reported that they had experienced a number (but not all) of these immediate adverse effects:

*Cos my jaw goes absolutely mental. It screws up. It clicks every time I move it. Everybody always comments on my mouth*. [Interview [5]]

*The fact that you can't urinate. I don't know about anybody else, but I can't go to the bathroom*. [Interview [6]]

A smaller number of young people had also suffered negative emotional or psychological effects whilst intoxicated. In contrast to the physical effects noted above, these were generally reported as being rare occurrences that did not happen on a regular basis. They included experiencing feelings of tension, agitation, anxiety, or paranoia. When this occurred, most young people reported that they withdrew from social interactions:

*I find party pills, you get times particularly for me towards the end of the night in particular, like the more like twisted euphoric party pills with the TMVP *[TFMPP]* stuff. They're like, get moments of real sort of withdrawn headspace where you just sort of like feel a bit funny and you don't want to socialise and your sort of thoughts get a bit weird*. [Interview [11]]

#### After-effects ('come-down')

Adverse effects experienced during the latter stages of the effects of BZP-party pills use were widely reported. These occurred in the hours and, in some cases, days after taking the products. In particular, an inability to sleep and a lack of appetite were two key issues identified. Other adverse physical effects included: tiredness and sluggishness, headaches, sore or dry mouth or jaw, and an aching/shaking body:

*At the end of the night you don't really feel tired. Like your body feels tired. When you think about it all you want to do is lie down. Your body is exhausted but your mind keeps going. And yeah, you just feel terrible and shaky and weak. You close your eyes but you keep seeing things and your mind won't stop*. [Interview [24]]

In addition, during this period, many young people stated that they suffered a range of negative emotional or psychological effects, including feelings of depression, tension and anxiety. For some participants, these were present the day after using BZP-party pills, whereas for others they extended well into the following week.

As a result of the impact of the substances on physical and emotional health, many young people reported that they withdrew from social interactions, or that their work or study performance had been impaired in the day(s) following BZP-party pill use:

*Definitely the next night if I have to go to work, I can't work, I'm extremely slow. Everyone gets really pissed off. So, I try not to go to work the next day*. [Interview [7]]

*Sort of depression, but not really, it's like, you know, when a woman gets her period she just really starts nagging at people and stuff like that, it's more like that. It's hard to explain really. You don't feel really sad or anything. You just feel tired and want to go to sleep and other people come in and try and try to keep you awake and you're like 'fuck off man'*. [Interview [18]]

Most young people considered the 'comedown' period induced by BZP-party pills to be very unpleasant, and those who had experienced the use of other stimulants considered it to be worse than with illegal drugs such as ecstasy. Similarly, for the vast majority, alcohol hangovers were perceived to be more manageable than those induced by BZP-party pill use.

## Discussion

This research comprises an in-depth exploration of young people's use of substances containing BZP. Although mainly a New Zealand phenomenon, the issue is emergent in other countries. Young people appear to be the majority of users of these products [3] and thus these results provide a unique and novel insight into their use.

A number of limitations exist which should be borne in mind when interpreting our data. Qualitative studies are not designed to produce generalisable results and this, combined with the small sample size, means that the findings may not be representative of the overall population – or young people specifically. The research may have attracted only those with more 'extreme' experiences or views of the topic. In particular, some young people were aware of media reports of efforts to see the products banned and were pleased to be given the opportunity to contribute to this debate. Other potential limitations include the possible clustering of experiences due to conducting friendship groups/interviews and the possibility of young people exaggerating their stories. Furthermore, a common characteristic of the BZP-party pill users interviewed was that they appeared to be a high functioning group, in that most were studying or employed, and they were articulate, punctual and reliable with respect to their participation in the study. This could be a product of the recruitment strategy, or may support previous claims that drug users from the dance party culture – with which BZP-party pills are strongly associated in New Zealand – are not as economically and socially marginalised as other regular drug misusers [17,18].

The study has revealed BZP-party pills fulfilling a range of functions for young people, with the products being used in a variety of situations and social settings – and has confirmed anecdotes that primarily linked the use of these substances with the dance party culture for the purpose of additional energy and enhancement of socialisation. Other uses have also been identified in this research, including BZP-party pills' role as a study aid, and fulfilling a weight loss function. Patterns of BZP-party pill use appear to be similar to usual patterns of substance use amongst young people in this age range. For example, experimentation is commonplace, much learning occurs through 'trial and error', levels of use fluctuate throughout the year, and mixing a range of substances (both legal and illegal) is common [19-21]. The perceived benefits of BZP-party pill use, such as increased energy and confidence, and enhanced sociability have been reported in other studies of illegal drug use [22,23].

The apparent trend with regard to young people reducing their frequency of use of BZP-party pills, as well as amount across a single drug-taking occasion, implies that use of BZP-party pills may be a phase of experimentation. Whilst this hypothesis has also been discussed in the media by individuals working in the health sector [24] it clearly requires further exploration. Regardless of whether young people go on to use BZP-party pills on a more regular basis, novice users should be a key target for harm reduction interventions, given the potentially risky behaviours they appear to engage in when trying the products for the first time (e.g. taking large amounts) and their reliance on (sometimes unsafe) advice from friends.

Given its stimulant effects, the way in which the products are marketed, and the cost of MDMA in New Zealand relative to BZP-party pills, it is perhaps not surprising that the substances are being used in similar ways to those seen amongst users of ecstasy in other countries; for example, prolonged periods of dancing in crowded and heated conditions, use to assist with weight loss, and the use of other substances to manage the 'comedown' period [12,22,25]. In addition, there was some commonality between the substances in terms of the negative effects identified, including mood fluctuations, broken sleep, lethargy and depression in the period after taking the drug [25-28]. This would suggest that, in the absence of research evidence about BZP-party pills, harm reduction messages from the party drug culture may be extrapolated to meet the needs of BZP-party pill users. This may include the importance of keeping close with trusted friends, taking 'time out' during long periods of dancing, and monitoring fluid levels and food intake, both when taking the substances and in the period after the effects of the drugs have worn off.

The research has identified a range of negative effects reported by BZP-party pill users. Some of the negative effects identified, such as tachycardia, can almost certainly be attributed to the pharmacological effects of BZP. However, many others (e.g. tiredness and lethargy) may be partly due to BZP's effect on sleep, combined with the effect of strenuous dancing for extended periods. In addition, the immediate and delayed combined physical and psychological effects of large quantities of alcohol or other substances combined with BZP-party pills are unknown. Clearly, further investigation is necessary to identify which effects specifically relate to the chemicals in BZP-party pills, and which are a by-product of associated behaviour and activities. However, in either case, issues such as sleep deprivation are important when considering young people given the links between sleep loss and reduced academic performance, car accidents, and gastrointestinal problems [29,30]. In addition, given the short duration in which BZP-party pills have been in existence, unlike other party drugs such as ecstasy, any longer-term effects of the substances are yet to be observed.

This study found limited evidence of significant short term harms having been experienced. Again, this may be an artefact of the make-up of our sample, being a relatively 'high- functioning' group. Despite this, the study has provided evidence that some young people appear to be participating in a range of risky behaviours with regard to BZP- party pills, including mixing substances, driving whilst under the influence of BZP-party pills, and taking larger numbers of BZP-party pills than recommended. These risky behaviours are not specific to BZP-party pills, and mimic behaviours seen amongst those who consume alcohol and other drugs [12,31]. Although there is currently no evidence specifically on the impact of BZP-party pills on driving, associated issues such as driver fatigue for those driving home after a full night of dancing and no sleep could be highlighted to users. Other research has identified that a lack of alternative transport options and cost have contributed to the reasons for people driving after using drugs [31]. This is likely to be the case for our youth sample, given their younger age and New Zealand's traditionally limited public transport systems.

The results raise a number of issues for harm reduction, in respect to the effects of use, context of use and potential risky behaviours as outlined above. The current, uncertain future of these products does not negate the need to provide accurate and appropriate harm reduction messages to young people and to explore environmental harm reduction issues. Even if ultimately banned, it is likely that similar products will emerge on the licit market. Moreover, BZP might become available illegally, or it may also emerge mixed in with other substances – evidence for which currently exists [32].

## Conclusion

This study is the first to explore issues around young people's use of BZP-party pills. Our findings suggest that young people in this study were not suffering excessive or dangerous adverse effects, despite reports of such events in other studies. However, potentially risky use of these products raises the issue of the need for developing harm reduction interventions. Information provision on issues such as not mixing with alcohol and other drugs, limiting amount taken, potential negative effects of the drugs and risky drug-taking contextual information may play a part in reducing the harm associated with use of BZP-party pills.

## Competing interests

The author(s) declare that they have no competing interests.

## Authors' contributions

RB participated in the study design, conducted and managed data collection and analysis, drafted the manuscript and edited the manuscript.

JS conceived the study, participated in its design and analysis, drafted the manuscript and edited the manuscript

Both authors read and approved the final manuscript.

## Appendix

^i ^Youth development 1) is shaped by the 'big picture'; 2) is about young people being connected; 3) is based on a consistent strengths-based approach; 4) happens through quality relationships; 5) is triggered when young people fully participate; 6) needs good information.

^ii ^A youth development approach would also ideally include 'member checking' by young people during the analysis stage of research in order to provide feedback on findings. Due to time constraints, this was not undertaken and is recognised as a limitation of the study.

^iii ^Due to confidentiality concerns, one participant requested that the interview was not audio-taped. In order that the data was still recorded, the researcher took hand-written notes during the interview.

^iv ^No recommended dose actually exists, as these are not medicinal products.

^v ^Use of nitrous oxide was also mentioned by several people during the interview/group discussions, although not noted in the pre-group/interview questionnaire.
